# Sport at the World Festival of Youth and Students: Between Olympic Ideals and Socialist Internationalism

**DOI:** 10.3389/fspor.2020.567095

**Published:** 2020-11-12

**Authors:** Lidia Lesnykh

**Affiliations:** Faculté de Sciences Sociales et Politiques, Institute of Sports science, University of Lausanne, Lausanne, Switzerland

**Keywords:** youth and students, student sports, university sports, soviet sports, sports and communism, festival of youth, socialist sport, socialist internationalism

## Abstract

During the first decade of the Cold War, the communist-sponsored World Festivals of Youth and Students included a program of international sports events that provided elite athletes with a self-standing arena of international competition. They also encouraged mass participation in sports, without social, racial, or political discrimination, thereby implicitly questioning elitism in sport. The present paper argues that through the World Festivals of Youth and Students, the Soviet Union harnessed the universal language of sport as a tool of cultural diplomacy with which to expand develop an international socialist sports youth network. The Festival sporting events represented an alternative model of international sport, run in parallel to the Olympics, whose ideals of peace, friendship, and mutual understanding they shared.

## Introduction

“Regardless of the size of the teams, whether they have won or not, the athletes form a big family under the Olympic motto: ‘Friendship between athletes strengthens the friendship of peoples^1^.”’ Although these lines could be mistaken for an extract from Pierre de Coubertin's writings, they actually had a very different origin. In fact, they summarized the Soviet sport ideology underlying the new concept of international sports competitions the Soviet Union created Olympics in the early Cold War. The Festival and its associated sporting events, both of which fit the definition of a “mega-event^2^,” pursued an original set of goals that combined the aims of Soviet cultural diplomacy and Coubertin's Olympic ideal. The present study examines to which extent the Festival's sports competitions can be considered an alternative platform for sports internationalism driven by Moscow during the Cold War.

Sports were a major component of the World Festival of Youth and Students (hereinafter referred to as “the Festival”), which was launched in 1947 and hosted by a different Eastern European capital city in the summer of odd-numbered years. Bringing together thousands of young people, they were initially conceived as a forum for promoting cultural exchanges and mutual understanding, but quickly became a tool of Stalinist propaganda abroad. The program included cultural, political, and educational events, as well as international sports events. It was drawn up at by an International Preparatory Committee under the coordination of Komsomol and the Communist Party of the Soviet Union^3^. In terms of their ceremonies, number of participants, and sports, the sports events were comparable to the Olympics, whose ideals they claimed to share, while advertising the achievements of the socialist system.

This paper examines the elite and grassroots sports events held as part of the Festival between 1947 and 1957. Grassroots sports events were held both before (“in honor”) and during the Festival. The elite-level competitions were the X and XI World University Games, hosted by Budapest (1949) and East Berlin (1951), respectively, and the I-III International Friendly Youth Sports Games, hosted by Bucharest (1953), Warsaw (1955), and Moscow (1957). Although the elite-level events were run by two communist-sponsored organizations founded immediately after World War II–the International Union of Students (IUS) and World Federation of Democratic Youth (WFDY)–, they had different origins. The World University Games were rooted in the French universalism of the early 1920s and were open only to students, whereas the Friendly Games were launched as part of the Festival in order to offer all young athletes, whether or not they were students, an opportunity to do sports without social, political, or racial discrimination. The Festival's sports events aimed at democratizing sport and were strongly tailored to the Soviet Union's cultural diplomacy purposes, enabling it not only to promote the regime, but especially to create a new arena for sports socialist internationalism^4^. They actually illustrated of how two rival socioeconomic models promoted their universalisms and internationalisms on a global scale with an important participation of the Third World^5^. These events also helped to defend “sports universalism in front of the social division^6^.”

According to Koivunen (2013), the aim of the Festival's organizers was “to conquer a field that still did not have a dominant leader or patron: youth^7^.” In other words, it was to occupy the domain of youth sports, which had not yet been appropriated by the International Olympic Committee (IOC) and the international sport federations, rather than to become a rival to the IOC^8^. Koivunen's pioneering research, based on large volumes of material from Soviet archives, examines the Festival as an instrument of Soviet cultural diplomacy and is a key reference for the present study. In the context of the present study, her thesis' title, “Performing peace and friendship,” takes on a double meaning, in that “performance” may also be considered to refer to sporting achievements. Koivunen borrowed Caute's (2003) concept of “Cultural Olympics^9^,” in which sports were an important soft power tool in the Cold War competition between the great powers. She also referred to Keys (2006), who pointed out the strong similarities between the Festival and the Olympics, most notably their ceremonial aspects, their regular and recurrent nature, and their “universalism going beyond nationalism^10^.” Second, she emphasized the Festival's link with both the mass cultural events of the 1930s and the World Fairs, which were created to highlight modernity and traditions^11^. I apply the term “cultural diplomacy” in harmony with the works of Koivunen's who considers it better adapted to the idea of cultural exchange. Gillabert confirms of “cultural diplomacy” in more linked to the cultural policy and the actors of the cultural field,” while “public diplomacy” corresponds “to the use of political communication and of cultural content as its tool^12^.” In fact, recent Russian studies design Festival as a tool of public diplomacy^13^. Finally, the Festival sports events inevitably embed in “sports diplomacy” as they participate in the Soviet foreign relations in this field. The literature review by Clastres (2020) underlines that the recent scholarship invites to reassess the contribution of sports to the international relations, both in link with *Realpolitik* and *soft power*^14^.

As Kotek (1998) showed in his seminal study of youth and student organizations during the Cold War, the international students and youth movement came to be dominated by communists in the late 1930s^15^. As a result, the sports competitions run by the IUS and WFDY were largely vehicles for communist cultural diplomacy. This premise is supported by recent research by sports historians. For example, Schiller (2019) shed the light on the institutional and national specificities of the X Festival in Berlin and the political use made of its sports events^16^, and Parks (2013) attributed the rise of Soviet sport and its international integration to the efforts of a “bureaucratic” element and its relations with the IOC and sport federations^17^. She argued that this task became easier in the context of peaceful coexistence and détente, because peace and friendship were a central part of this discourse. This “process of melding Soviet and Olympic ideals^18^” had already been noted by Riordan (1974), who also maintained that “the USSR attempted to impose itself in international sports by creating new institutions instead of integrating the existing ones^19^.” Parks (2014) pointed out the importance of the developing nations in Soviet sports diplomacy^20^. In addition to these references, the works of Prozumensikov (2004) based on the Soviet archives offered a deep analysis on the state participation in of the Soviet sports relations. Examining the Soviet sports system from an institutional perspective, Dufraisse (2019) suggested that the Soviet sporting champion could be regarded as the product of an engineering laboratory^21^. Hence, the Soviet sportsman was a “new man,” created in the 1930s, who acted as an ambassador for Soviet values and contributed to the internationalization of Soviet sport^22^. Although Dufraisse only alluded to the Festival, he emphasized the importance of youth in post-Stalinist propaganda. His findings actually echo in the part of this paper dedicated to the profiles of some young athletes^23^. However, despite the extensive scholarship, very few studies have focused on sport at the World Festival of Youth and Students.

## Materials and Methods

This topic lies at the intersection of sports history and the history of youth and students. From a broader perspective, it touches upon the history of cultural diplomacy, the Cold War, internationalism, pacifism, and international relations, as well as colonial and post-colonial history. The research draws material of Russian public archives, archives of the International Olympic Committee, as well as the collections from the International Institute of Social History (IISH) in Amsterdam. It also relies on press articles from the USSR, France, Italy, and Switzerland. The goal of the archival research in the Soviet Archives was to understand which aspects of the Festival sports program were particularly discussed at the superior level of Soviet state and which other authorities were implied in this process. The archival material reveals the multitude of interactions and the complexity of the decision-making process. It encompasses reports of delegations, correspondence, and circular letters produced or received by the Committee for Physical Culture and Sports, the Foreign Policy Department of Central Committee of the Party. The exchanges with Komsomol and in particular its leader Nikolaj Mihajlov represent a crucial part of this documentation. Even though archives are open to consultation, many of files are still not declassified, so some demands were refused due the secret character of the documentation^24^. These restrictions reduced the volume of accessible material. I could partly fill this gap by studying the Soviet press and official publications of the IUS, along with the WFDY found at Russian public libraries as well as the IISH. Secondly, the correspondence of the IOC leaders was an essential source for understanding of personal and institutional points of view. The IOC archives were necessary to take knowledge of the reaction (or of its absence) of its leaders to the introduction of sports to the Festival and the possible exchanges with other IOC members or other organizations on this topic. When analyzing the attitude of national and international sports institutions, it was necessary to reduce the list to the IOC and FISU. Finally, due to the important volume of archival materials, I studied press selectively. I particularly examined Soviet newspapers and magazines in order to assess how much attention sports press paid to the Festival events, and on the contrary, if sports deserved the interest in the newspaper of Komsomol. On the other hand, I had to strictly limit the number of titles of foreign newspapers. Since France and Italy were the two countries with strong communist parties, this explains the choice of *L'Unità* and *Le Monde* having different political perspectives.

Adopting a chronological approach enabled me to identify links between sport at the Festival and changes in Soviet foreign policy. I begin by examining the World University Games' role in enabling the Soviet Union to integrate the international sports movement during the late Stalinist period then show how sport at the Festival mirrored the Socialist bloc's overtures to the Third World. Finally, I look at Moscow's ambition to become one of the major international sports destinations.

## “It is Impossible to Imagine Youth Without Sport”: Sport'S Faltering Debut At the World Festival Of Youth and Students (Prague, 1947)

Sport was also an important part of the Festival's program, as the organizers considered it, by its very nature, to be a youthful activity^25^. Democratic and autocratic states had begun conjugating sport with youth in the 1920s−1930s, and this association was an important characteristic of physical education in early Soviet Russia^26^. The sports program of the Festival was run under the auspices of the host countries' sports authorities and youth communist organizations, but the Soviet Union's Sports Committee and Komsomol closely monitored preparations and offered recommendations. Around 17,000 young people from 17 countries, predominantly European, arrived in the Czechoslovak capital in August 1947 for the first Festival of Youth and Students for Peace and Friendship. The Festival's sports program appeared quite extensive and encouraged mass participation: “it offered to the guests the possibility to choose among the variety of entertainment activities, from football to ping-pong and chess. The competitions will arrive at the point culminating during the last week, when the best teams of all peoples will compete”^27^^,^^28^. Sport at the Prague Festival was open to all, subject to advance registration, and included 75 events^29^, ranging from elite individual and team sports, recreational sports, tests of strength, intellectual competitions, and sports demonstrations. According to the events' start sheets, 103 teams from 27 countries, took part, with 1337 athletes competing against their foreign comrades in 13 sports common either for elites, or for working class: swimming, tennis, table tennis, chess, athletics, basketball, volleyball, shooting, wrestling, boxing, weight lifting, cycling, and rugby^30^.

Due to gaps in the archives, it has so far been impossible to find detailed information on selection criteria for athletes. Given that attracting as many participants as possible was one of the Festival's goals, restrictions relating to age, nationality, or club membership are unlikely to have been imposed. Many athletes participated as members of youth or sports associations. For example, the Italian athletics federation (FIDAL) entered a track-and-field team, Italy's volleyball confederation entered a volleyball team, and a left-leaning student organization (Centro Universitario Democratico Italiano) chose athletes in other sports^31^. Soviet athletes competed in six sports (athletics, gymnastics, swimming, weightlifting, volleyball, and basketball), making Prague one of the few occasions on which the Soviet Union competed abroad before becoming affiliated to the international sport federations and the IOC.

Indeed, after having missed from the “bourgeois” sports arena, the USSR affiliated several international sports federations in the late 1940s, and the International Olympic Committee (IOC) in 1951^32^. Furthermore, the USSR entered in contact with capitalist countries and the federations and sought to observe the Western athletes' training methods with an intention to enrich its own ones^33^. In this context, the promotion of regime abroad and establishing sports connections within the socialist bloc were clearly among the main reasons why the Central Committee of the Communist Party decided to send a team to Prague. According to a document sent to the (Komsomol) and labeled “top secret” (like many other documents relating to the Festival), the aim was “[to demonstrate] the achievements of the multinational Soviet Union in the fields of culture, art, and sport, the role of Soviet youth during the war and post-war reconstruction, and to demonstrate the state's care for its youth^34^.” Soviet athletes successfully accomplished this task. For example, the 10-person mixed gymnastics team performed brilliantly and were awarded both a Crystal Cup by the WFDY Executive Committee and the Miroslav Tyrš medal^35^, and a team from Leningrad won the volleyball tournament. The swimming team participated out of competition, as the country was not yet a member of the international swimming federation^36^. According to the report written in the best traditions of Soviet state, these performances “highlighted [Soviet athletes'] high moral qualities, their will to win and their great sporting mastery, and, at the same time the government's interest in developing education, culture and sports^37^.”

The number of sports on the program was quite impressive for this post-war period, especially as five of them–volleyball, rugby, tennis, table tennis, and chess–were not on the Olympic program^38^. Giving floor to the non-Olympic sports during the Festival was probably an attempt of rapprochement with the respective sports federations. Moreover, the approval from the other international federations was likely to be difficult and there was even a chance they would react negatively to the sudden appearance of an international multisport event in Eastern Europe just before the London Olympics. It is also noteworthy, that in 1948 the WFDY's magazine expressed hostile attitude toward the IOC and described international sport institutions as the “bourgeois” fruits of capitalism^39^. It accused “the dark forces of imperialism [of compromising the true character of the Olympic Games, which] could become one of the best manifestations of peace and friendship^40^.” The link between sport, youth, and peace clearly alluded to the three components of the Soviet sporting universalism advocated by the Festival in contrast to the Western sport. Although Moscow's plans to develop a youth sports movement were not explicit for the West, the IOC's leaders, who were already reluctant to the communist participation in international sport, quickly expressed their concerns^41^. In December 1948, the IOC's chancellor Otto Mayer received two telephone calls from “behind the iron curtain^42^.” The Czechoslovak and Polish Legations in Switzerland had unexpectedly requested information about the IOC's plans with respect to youth. The IOC's president Sigfrid Edström immediately suggested that “Moscow [was] organizing something^43^.” These two calls actually seem to have been linked to preparations for the sports events at the second Festival in Budapest, the following August, which had been chosen to host the 1949 World University Games.

## The World University Games at the Festival: Sport in the Students' Cold War (1949–1951)

The fact to combine the World University Summer Games with the Festival as of 1949 made it possible to increase collaboration between the WFDY and IUS, and therefore facilitated the goal of bringing together the world's youth through sport. However, the context in which the 1949 and 1951 Festivals took place was far from favorable to peaceful cooperation. The institutional split in the university sports movement since the creation of the International University Sports Federation (FISU) by the Western countries in early 1949 was one of concrete consequences of Cold War: the introduction of the Marshall Plan, the dispute between Tito and Stalin, and the outbreak of the Korean War directly impacted student and youth movements^44^. Increased tension between the blocs since the autumn of 1947 also led to substantial changes in the rhetoric of the 1949 and 1951 Festivals, which evolved from the 1947 Festival's focus on universalism and the struggle against abstract enemies of peace to the 1949 event's condemnation of the reactionary forces of the United States and Great Britain^45^. And although both Prague 1947 and Budapest 1949 had showcased the Soviet Union's cultural and sporting achievements, by Berlin 1951 the focus was on demonstrating the USSR's geopolitical power^46^.

Moreover, the international sports scene was also changing. In the spring of 1951, the Soviet national Olympic committee was admitted into the Olympic movement. The expansion of sporting exchanges announced at the 1951 World Peace Congress in Vienna “had to allow the best Soviet athletes to spread the notion of peace and friendship between peoples^47^.” These events greatly increased the importance of the University Games in Berlin for the Soviet Union, as they provided a major proving ground for Soviet athletes in international competitions. They were also crucial for the International Union of Students because the creation of FISU in response politicization of the University Games had upset the delicate balance of power within it^48^. Although the FISU was a small organization, it was based in Switzerland and was therefore better placed to please the IOC and international sports federations. At the same time, incorporating the university games into the Festival jeopardized the Soviet Union's influence over international student sport and the event's prestige.

The integration of the World University Games to the Festival meant the event could no longer be reserved for elite student athletes and would have to embrace a more inclusive logic centered round youth cooperation. The Festival helped popularize the university games among youth and students from more countries. As well as being the second largest multisport event after the Olympics, the university games had a long history and an excellent reputation within international sport. Consequently, they were able to attract a large number of countries from outside the Olympic movement (countries within the Soviet bloc and developing countries). The Soviet press often compared performances at the university games with those achieved at the Olympics, and foreign communist newspapers claimed that “the presence of many Olympic champions and world record-breakers gives the event the character of a true Olympiad^49^.” In fact, the two events were not only comparable in scale and sporting performance, they also had similar ceremonies.

Although the Festival and university games had separate opening ceremonies, both ceremonies usually included sporting elements. A few weeks before the Budapest 1949 opening ceremony, peace relays, in which runners carried WFDY flags, started in several European countries (Bulgaria, England, Norway, France, Belgium, Netherlands). At the same time, youth organizations in Austria and Romania held, respectively, bicycle and motorbike races^50^. On August 14, 1949, 10,400 festival delegates from 82 countries paraded into Budapest's Ujpest stadium^51^. The Soviet Union's young delegates, who were dressed in white as a symbol of peace, were warmly welcomed in the speeches addressed to Stalin and the Komsomol^52^. The university games opened the following day with a friendly football match at the Ferenzvaros Stadium featuring France and North Korea, the winners of the football competitions at the Prague Festival in 1947. Sixteen countries, represented by 934 athletes, including 303 from Hungary, the host country, and 117 from France, took part in the event^53^. North Korea's delegation of 24 athletes was bigger than those of Mongolia, Scotland, Austria, Belgium, and Finland, taken together.

The next Festival in 1951 in Berlin took place in “crucial time of diplomatic battles for the GDR's international legitimacy^54^.” It was described as a “great cultural, artistic, and sports event^55^,” whose opening ceremony was described by Lausanne newspaper as “a great style show, in perfect alignment with the powerful propaganda spread every day in East Berlin^56^.” As in Budapest, the university games, held from August 6 to 15, were opened separately. The games were opened not by the GDR's president, Wilhelm Pieck, but by the first secretary, Walter Ulbricht, who used his opening speech, given in the stadium named after him, to stress the games' importance in promoting peace^57^. The traditional oath, pronounced by Georg Frister, a “master of sport^58^” and holder of the East German triple jump record, was adapted slightly so it resonated better with the festival rhetoric. Thus, the expressions “honest combatant” and “contribute to […] the strengthening of mutual understanding among students from all over the world^59^,” which were typical of the Festival's ponderous pacifist discourse, replaced “the glory of sport,” which had been part of the oath since it was introduced in the 1920s.

In line with the IUS's objective of making Berlin 1951 the largest and most representative Games ever held, a new record was set in terms of participation, with 2,000 athletes from 40 nations taking part in the sports events, out of the 26,000 delegates from 105 nations who attended the Festival^60^. The criteria for taking part made it relatively easy to achieve this typically Soviet, maximalist goal, as the regulations sent to national sports organizations in May 1951 did not require athletes to be affiliated to a student sports association and allowed high school students who had reached the age of majority to take part. The Soviet newspaper *Sovetskij sport* noted its impression that “sport has an even more important place in Berlin^61^” and that “holding the Games during the Festival [.] would be a wonderful opportunity for student athletes to compete in front of an audience of young people and students from more than 80 countries^62^.” Most of these young people were from Eastern Europe, largely due to the split in the university sports movement and the reluctance of Western student sports associations to send their athletes to the games. In addition, Western countries, such as Italy^63^, were not adverse to placing obstacles in the path of students to prevent them taking part in the Festival. Such instances of visas being refused or problems crossing the French, Swiss, Spanish, or Italian borders were widely reported in Soviet newspapers and magazines. According to *L'Unità*, the NOCs of numerous capitalist states “sabotaged^64^” the games in order to prevent athletes taking part in this “proving ground” for the Helsinki Olympics.

Budapest and Berlin were key competitions for the Soviet team before its test in international sport at the 1952 Olympic Games in Helsinki. University games showcase of the advantages of the Soviet sports system, so that Soviet sports authorities needed to organize a meticulous training program and select very carefully the athletes they sent to the Festivals. Nevertheless, choosing the Festivals for the Soviet team's international debut after Stalin's refusal to send a team to the London Olympics^65^, meant that their athletes would not compete against the Americans, who refused to participate in the communist-run event. Despite the Soviet athletes' numerous victories in Budapest and Berlin, the experience showed that many aspects of their training would have to be improved if the team was to be successful at the highest levels. Since the experience of Budapest and Berlin seemed to prove that the Festival was an appropriate platform to achieve Soviet sports diplomacy goals, World University Games were replaced by a new event open to all youth and thus, possibly worrying the IOC leaders.

## “None of Our Business”: the Festival and the IOC'S Leaders

The 1953 edition of the Festival saw the introduction of a modified sports program, in which the International Friendly Youth Games replaced the World University Games. While the latter were already a recognized event in Western Europe and around the world, the new competitions associated with the Festivals since 1953 would have to build a reputation from zero and stake their place in the international sport. The first edition of the Friendly Games coincided with increased openness to the outside world on two fronts, politics and sport. From a political perspective, Stalin's death in March 1953 had been followed by changes in Soviet foreign policy, including greater openness to countries outside the communist bloc. Moreover, 1951–1956 was a crucial period in forming a new international identity for Soviet sport. Thus, the Friendly Games most probably reflected the ambition of the Soviet authorities to test the new, more accessible international multisport event, since limiting the competitions only to students was incoherent with promoting socialist values among all youth. However, the Friendly Games could not expect to gain automatic recognition from international sports federations and the IOC and need to be intensively promoted: the credibility of the Festival's sports competitions was a major concern for the WFDY. It was important for the sports events to be seen to follow internationally recognized rules in order to avoid similar suspicions of cheating or bias to those leveled at the judges of the Festival's artistic contests. Even though the Friendly Games applied international sport federation regulations and the regulations on amateurism, as defined by the IOC, it did not prevent from participation of state-sponsored professional athletes, as it was common for the socialist countries.

The games' organizers built trust and closer relations with the IOC and the international federations by inviting their leaders to the event^66^. This approach had already been used at the university games in Budapest and Berlin for fear that international federations would recognize the rival FISU as the main student sports organization. In fact, the FISU's International University Summer and Winter Sports Weeks launched in 1949 were a direct threat to the World University Games run by the IUS. Funding was not an issue for the IUS and WFDY, as support from the Soviet Union ensured they had greater financial and human resources than the FISU supplied only by the membership fees. The real problem they actually faced was that the FISU was a purely Western institution and therefore more likely to appeal to international federations and the IOC. In a personal letter to Sigfrid Edström and the IOC's secretariat, the World University Games organizing committee evoked the “great tradition of university sport” and highlighted the fact that the games were founded “on the basis of the Olympic concept in order to contribute to the understanding and friendship of peoples^67^.” The letter was undoubtedly intended to promote the IUS's World University Games ahead of the FISU's Sports Week and thereby help address a worry expressed by the Soviet authorities: “there is a danger that international sports federations will recognize the FISU's meetings as student championships^68^.” Finally, the International Basketball Federation's secretary general, William Jones, attended the games on his own initiative to clarify the situation in student sport. The IUS's invitation for IOC officials to come to East Berlin came just 2 months after the IOC had recognized West Germany's national Olympic committee and at a time when the only international federations to recognize East Germany were basketball and chess, which meant athletes in other sports who competed against East German athletes risked being unable to participate in future competitions.

During this split in the university sports movement, which lasted from 1949 to 1957, the FISU also asked the IOC for advice, both to obtain moral support and to free itself from having to make the highly political decision about which countries to admit as members^69^. Although the University Games were no longer part of the Festival, the Friendly Games, which were held from 1953 to 1957, were also for students. FISU's secretary general, Carl Schneiter, warned Otto Mayer that the Games were “a pure communist propaganda^70^” exercise that threatened sports neutrality. He assumed that some countries were planning to go to the Friendly Games in Warsaw in 1955 but not to the 1956 Olympics in Melbourne, partly because of the cost, but also to avoid the antagonism between the USSR and the United States. His fears were confirmed, he believed, by an article in the Belgian newspaper Hermès, according to which the Friendly Games were “a clear attempt to take away the meaning of the Olympic Games and, if possible, take their place^71^.” According to the article, the Festival's organizers were trying to attract to Warsaw countries which were unsure about going to Melbourne, and concluded: “It is not because the international situation goes through periodic phases of détente that the West should lend itself to participating in a communist organization […] however naive Westerners may be^72^.”

Nonetheless, Mayer did not openly share Schneiter's concerns: “I do not believe, however, that the danger is as great as you assume. Sport is still in the hands of the International Federations and as long as it is the case, it will be very well-governed. Your cry of alarm deserves to be dealt with, but it is not to the IOC to do so. It is rather the responsibility of the International Federations^73^.” Mayer's reply was characteristic of the IOC's strategy of avoiding issues outside the Olympic movement and inviting the federations to deal with them^74^. However, a few months later, he sent quite an ardent letter to the IOC's president, Avery Brundage, about a report he had received from the International Rowing Federation's president Gaston Mülleg. “This is a sort of copy of the Olympic Games, with the difference, that 27,000 athletes were present at the Parade of the Opening Ceremony! […] There is no Olympic flag at all, and he [Mülleg] did not hear a single word about politics. […] Besides that, they had wonderful receptions, wonderful food (as much champagne, caviar as they wanted) and everything free of charge^75^.” Mayer was aware of the political nature of the event and agreed with Mülleg, who compared the Festival with “what Hitler had done in Germany before the war,” referring to the propaganda campaign of 1936 Summer Olympics. Last but not least, correspondence between Mayer and Brundage reveals the IOC's leaders ambiguous attitude to the Festival, given that the attempts to build a socialist international sports system separately from the Olympic movement existed before the war. It also appears that Mayer's interest in the issue not just due to his capacity as IOC chancellor, as his son was extremely interested in taking part in the Festival, attracted, partly, by the low registration fee, which included meals and accommodation. Although Mayer realized his son was “far from being a communist^76^,” and only wanted to go to Bucharest to satisfy his curiosity about the event, Mayer forbid him from going. Other letters show that Edström was also aware of the event's political nature^77^, unlike Brundage, who replied: “this, of course, is none of our business but it might be placed on the agenda of our meeting with the International Federations,” adding: “There can be no objection unless they start to mix politics with sport^78^.”

## See the Stars Up Close: Using Olympic Champions to Promote the Friendly Games

In parallel to the sport leaders' recognition, the Friendly Games organizers needed to gain a reputation among athletes and countries as well as to form positive image in public opinion. The media coverage was not limited to the official festival publications, socialist countries', and foreign left newspapers. They provided interviews with famous athletes invited to compete from all over the world to increase the games' prestige. The Western general and specialized media also reported on the Festival sports events, without citing interviews but focusing on the results.

The International Friendly Youth Games were scheduled so they took place twice between two editions of the Olympic Games. For many athletes, the 4-year gap between Olympic Games was too long and the Friendly Games provided an opportunity to compete against their Olympic and Championships opponents every 2 years. The shorter intervals between Friendly Games and their very flexible (sometimes non-existent) qualifying system made them very attractive to athletes who wanted to test themselves against world-class opposition or when they were unlikely to have a chance of competing in the Olympics. Finally, the opportunity to visit Eastern Europe's capital cities was just as appealing to athletes as it was to participants in other aspects of the Festival. However, to achieve the aim of becoming “contests of the best^79^,” the organizing committee used to invite famous athletes from both East and West. Athletes from the West were not paid for taking part, so they would not lose their amateur status, but the low registration fees and cheap accommodation meant that the cost of competing was unlikely to deter most of them. The presence of international athletes who had accepted an invitation to compete was sure to increase the event's prestige, and the desire to meet and/or compete against these champions was highly motivating for other potential competitors. Thus, the Friendly Games would become the place to be: where else could you compete against Emil Zatopek and dozens of other famous athletes? The emergence of the Soviet Union as an elite sporting nation and the increasing number international medals being won by the country's athletes was a further motivation. Indeed, Soviet and Eastern bloc athletes were beginning to catch the attention of sports fans around the world thanks to their performances in international competitions, most notably the Olympic Games. After rapidly raising standards between 1951 and 1955, the Soviet Union was able to send a truly world-class team to the 1956 Melbourne Olympics, where, for the first, the Soviet Union won more medals than the United States.

Zatopek undoubtedly attracted a lot of public attention. The French *Le Monde*, which reported results from the Friendly Games, mentioned Zatopek more frequently than any other athlete^80^ and a photo of Zatopek featured in the Swiss Festival Committee's brochure under the heading: “Will Emil Zatopek set a new world record?^81^” As *Komsomol'skaja pravda* noted, several Olympic champions said they were keen to compete in the Friendly Games. They included the Jamaican sprinters Rhoden and McKenley, the Hungarian hammer thrower Csermak, and the Zatopek couple^82^. Other athletes who accepted invitations to compete included the Australian sprinter Shirley Strickland, the Brazilian long jumper Adhemar Ferreira da Silva, the Romanian table tennis player Angelika Rozeanu, the French swimmer Aldo Eminente, the Hungarian long jumper Olga Gyarmati, and the Soviet discus thrower Nina Romashkova (Ponomaryova) and long-distance runner Vladimir Kuts. Interestingly, for rising stars such as the Algerian-French marathon runner Alain Mimoun and the American javelin thrower Dave Stephens, taking part in the Friendly Games proved to be a step on the way to Olympic glory in Melbourne.

Another important way of increasing the games' renown was through publishing interviews with athletes, who were quoted as expressing great enthusiasm for meeting Soviet “stars” and the spectacular progress they had made. The veracity of many of these interviews appears doubtful, given their propensity to mention the same subjects, such as the victory of friendship and peace being the most important result of the competitions, while noting the high standard of the competitions in general. Many of the supposed interviewees were national, international, or Olympic champions. For example, Brazil's double gold medal-winning triple jumper and student, Adhemar Ferreira Da Silva, said that he had “checked the list of participants” before accepting the invitation, and that he had decided to compete and “put everything on the line” after seeing the high standard of the field^83^. For the Hungarian discus thrower Ferenc Klics, who competed in four Olympic Games between 1948 and 1960, “[athletes] will only be able to gain popular recognition when they understand that sport is closely linked to the great struggle for peace^84^,” while Danish wrestling champion Eigil Iohansen looked forward to hearing the best athletes coming together “in a friendly competition,” which he saw as “an opportunity to renew the friendship of young people from all countries^85^.” Repetitive references to “all countries” underlined the intention of the USSR to democratize sport in in parallel to the internationalist ambition of the Festival sports events. It concerned most notably Third World countries, including those whose national bodies had not yet been recognized by the IOC and international federations. In this respect, it is noteworthy that the nationality of interviewees changed in line with the shifts in Soviet foreign policy: most interviewees at early editions of the Friendly Games were from Latin America, Africa, Asia, Scandinavia, and Finland; whereas later editions also saw interviews with Americans, Australians, and New Zealanders. As well as reflecting the Soviet Union's evolving geopolitical focus, interviewing people from a wider range of countries highlighted the event's global character, thereby increasing its legitimacy^86^.

## Mind and Body, Peace and Friendship: Olympic Ideals, Soviet Style

Referring to peace, friendship, and solidarity was an effective way of promoting the communist and sports values without directly referring to communism as such^87^. At the same time, as asserts Toby Rider (2018), “the Soviet regime tried to create the impression that the noble aims of its sports model were representative of the virtuous goals of the Soviet state^88^.” Since the 1940s, the heads of the Soviet Union's sports organizations had capitalized on the similarities between “aspects of Olympic philosophy [and] Marxist-Leninist ideas of mass participation in sport, promoting physical education for all, and peace and friendship between nations^89^.” This transition from criticizing the bourgeois Olympic institution to progressively embracing the values of the Olympic movement marked a shift in the way the WFDY ran the youth sports movement. Its aim was to consolidate the symbolic basis of youth sport by establishing links with ideals of friendship and peace proper to Olympism.

The integration of the USSR to the IOC and to the international federations progressively changed the tone of publications after Stalin. In 1956, the Olympic Games became a popular topic in the WFDY's magazine, World Youth, which had tended not to write about sport, while the IUS and the Soviet press published innumerable articles in favor of the Olympic tradition. For example, in the run up to the games in Melbourne, *Fizkul'tura i sport* published a two-page article on the history of the Olympic Games^90^. The goal was to show the extent to which the games associated with the Festival helped continue the Olympic tradition. In an interview ran by World Youth, the WFDY's secretary for sport, Hungary's Mihaly Biro, stressed the importance of the “physical fulfillment of young people [who had to] stimulate the development of friendship between the youth of different countries and [educate] them in the spirit of the Olympic ideals^91^.” Another article highlighted the importance of the Olympic Games “for all mankind and the younger generation [since they contribute] to expanding the bonds of friendship between peoples and the youth of different countries and strengthening mutual understanding^92^.” According to the Soviet press, the Friendly Games in Warsaw “had undoubtedly contributed to popularizing sport and the Olympic ideal” and, “in terms of their scale, [were] equal to the Olympic Games^93^.” However, the Friendly Games and Olympic Games were not in competition; rather they “complemented each other [in such a way that the Friendly Games] became a ‘pre-Olympiad^94^.”’ Obviously, this frequent reference to the Olympics did not aim at promoting them *per se* but to underline the internationalization of the socialist sport.

Moreover, the idea of harmonious moral and physical development advocated by Coubertin was also promoted by Soviet sports ideology since the 1920s. In fact, many of the athletes who competed in the Friendly Games were students^95^. Poland's Janusz Sidło, a student at the Academy of Physical Education in Warsaw who competed in the 1954 World University Games and 1954 Athletics World Championships, noted the importance of maintaining a balance between studies and sport. Such a balance was achieved by, among others, Tamara Manina, a rising star of Russian gymnastics, who won two individual silver medals and team gold and bronze medals at the Melbourne Olympics (as well as gold and silver team medals at Tokyo 1964), as well as being both a student of physics and mathematics at the University of Leningrad, and a musician. Combining other commitments with their sporting achievements not only enabled athletes to show they were truly amateurs, the ability to succeed in several fields was trumpeted as showing the advantages of the socialist system and of its intercontinental expansion. For example, the young captain of a Chinese basketball team had begun life as a peasant and had learned to play basketball with the factory team at the steel plant at which he had found work when the communists came to power^96^. Finally, Soviet sports system frequently hailed the proletarian origins of many of its top athletes. Emil Zatopek's noted Soviet rival, Vladimir Kuts, who was frequently glorified in articles about the Festival, was a typical Soviet “sports hero,” who had risen from modest beginnings to become a champion in his sport^97^. This facet of the Soviet sports doctrine going hand-in-hand with its criticism of sports elitism, glorified the socialist educational system, which allowed to develop the personality and talents regardless the social origin.

## Democratizing Sport: Between Grassroots Sport and Globalist Ambitions

Having set “promoting sport among youth^98^” as its goal, the Festival's sports program aimed to encompass socialist internationalism on a societal scale and to democratize sport. In other words, the Festival helped to expand participation across social groups, thereby reflecting efforts made by the Soviet government to encourage people from all walks of life to take up some form of sport, whatever the level. Sports actually participated in creation of a “new man^99^” within and outside the Socialist bloc. Although grassroots sports events at the Budapest and Berlin Festivals had been low key and run in parallel to the University Games in order to encourage non-students to participate, they took on a whole new dimension as of 1953^100^. Sports events at Bucharest 1953 were, according to the Festival's regulations, open “to the youth of all countries” “with no limitation on age, social position, or political, and religious views [.]^101^,” while the competitions at Moscow 1957 were open to “individual athletes or teams, workers, students, and other sport clubs^102^.” This latter quotation neatly summarizes the alternative conception of sports meetings drawn up by the WFDY.

To ensure the sports program was inclusive and as a way of encouraging mass participation, numerous different events were held both before (“in honor of”) and during the Festival. The most important events were friendship tournaments and festival badge tests. Mass participation events were held in 13 or 14 individual and team sports, including some non-Olympics sports, during the 2-week Festival period. Around 2,000 athletes took part in these events. In addition, hundreds of local play-offs, and cross-country races, involving thousands of participants, were held during the months leading up to the main event. In many countries, including Italy and France, they were associated with existing events run by organizations close to the national communist party^103^. The WFDY and the IUS also tried to expand their influence outside Europe by holding sports meetings and camps. For example, Young Brazilians played in 208 friendship tournaments at the South-American Festival of Youth in Sao Paolo, even though the Brazilian government had banned them from taking part^104^, whereas several clubs in Algeria took part in the Festival Badge scheme^105^. The Festival Badge, which was awarded from 1953 to 1957, was designed to encourage all Festival delegates to test their sporting abilities. This voluntary awards system was inspired by the Soviet Union's GTO badge introduced in 1931 to recompense ordinary young men and women physical performance^106^. Participants were awarded a gold, silver, or bronze badge according to their performance on a series of athletic and gymnastic exercises. According to statistics for the Warsaw 1955 Festival, 16,000 young people from 57 countries took part in the Festival Badge program, which “incarnated the memories of friendship born on sports fields^107^.” In fact, the Festival Badge scheme, which was one of the most important programs for popularizing physical exercise and, of course, the Festival and communism, was also open to young people who could not travel to Europe, thereby enabling them to participate indirectly in the Festival. Badges were awarded in Indian villages, “where young men and women, lacking the weightlifting equipment needed for the competition, used bags filled with rice^108^.” The example illustrates that Soviet authorities employed the GTO-program among other methods to help the developing countries to modernize their national sporting culture. In addition, these local events sensitized youth to the different aspects of the Soviet life: for example, an Indian young lady from West Bengal was interested in “sports and cultural institutions” of the USSR^109^.

In parallel, individual athletes and delegations could improve their performances and reassess national training systems, especially in former colonies that had recently gained their independence. It was important not only to participate, but to learn: “athletes come to the Festival not only to fight for victory and compete with the strongest but to learn and accumulate experience^110^.” For example, a Chinese coach photographed famous athletes performing in order to help improve training methods in China, and a member of the Indian Field Hockey Association expressed his wish to spread his sport throughout the world and develop other sports in India in exchange^111^. Festival created a framework favorable for the improvement of results by providing up-and-coming athletes with the support they needed to realize their full potential^112^. For the Soviet Union, these events provided a useful political tool through which they could support newly independent countries and national liberation movements and helping them develop sporting culture among their peoples. Thus, the Festival's sports events were also intended to showcase the solidarity of the USSR and the socialist countries with the Global South and probably set the premises of Olympic Solidarity several years before the USSR suggested the idea to the IOC in 1961.

## The International Solidarity Fund: Conquering the Hearts and Minds in the Global South

The festival sports program was strongly linked to the geopolitical battle in the colonial and post-colonial world. This especially interested the Soviet Union willing to expand its sports influence in these countries in its self-appointed role as the “champion of anticolonialism^113^.” Despite Stalin's isolationist foreign policy, the IUS and WFDY had taken an interest in decolonization from their creation. These two organizations aimed at extending their influence beyond the communist bloc in order to “spread and advance the democratic ideology, in other words, help colonial students in their struggle for liberty and independence^114^.” The IUS had support in Asia, the Middle East, Africa, and Latin America, since it “unambivalently backed anti-colonial movements where emerging students' leaders could be found^115^.” References to developing countries were present but still relatively restrained during the late 1940s and early 1950s. For example, in an article relating to the 1949 Festival in Budapest, *Sovetskij sport* wrote: “it was difficult to imagine the enthusiasm with which the young men and women of the Hungarian People's Republic greeted youth ambassadors fighting against the colonial regime, against imperialism, for national independence, peace, and freedom^116^.” The rise of national liberation movements and the accelerating pace of decolonization increased the Soviet Union's interest in these regions during the 1950s, especially following the 1955 Bandung Conference. According to J. Parks, “bring[ing] more nations into the Olympic movement” had become an important target for the Soviet Union's new leadership^117^. The USSR worked to increase the African influence within the IOC, a process that paralleled the growth in the number of African national Olympic committees granted IOC membership between 1952 and 1960^118^. In the framework of the Festival, the idea of attracting students from countries fighting for or recently granted independence had been turned into a concrete policy by 1950. The International Preparatory Committee for the Berlin Festival created a solidarity fund to help delegates from distant countries attend, specifically young people from colonial and dependent countries from Latin America, Africa, the Middle East, and South-East Asia.

More than 700 delegates attended the Berlin Festival, some of whom also took part in the University Games^119^. World Youth stressed “the importance of the participation of athletes from colonial and dependent countries, as well as from Latin American countries^120^” at Berlin. According to the IUS's president, Joseph Grohman, the “sports activity [of the IUS] aid the struggle for democratic education and the provision of greater opportunities for students from all countries to practice sport^121^.” Several months before the Festival, richer countries began collecting money, via national solidarity committees, cultural and sports events, voluntary work, and donations, that could be used to help countries without the means to send athletes. For example, money from street concerts organized by young Swedes was used to pay for the Sudanese delegation's trip, young Finns volunteered to help young Senegalese delegates^122^, and young people from England and France held collections for delegates from Malaya and Kenya, and from Tunisia, respectively. According to statistics in the Soviet archives, Poland raised $50,000, China $35,000, Hungary $30,000, Romania $14,000, France $7,000, England $2,500, and Finland, Sweden, and Italy $1,500, each. However, the Soviet Union did not officially participate in the fundraising^123^.

Of course, the press and other documents focused mostly on politically unstable regions of geopolitical interest to the USSR, and the countries cited changed as Soviet interests evolved. Greater attention was being paid to distant countries as potential participants in the University Games (e.g., Brazil, Ecuador, Indonesia). India was one of favorite examples, as the Soviet Union had established diplomatic relations with the Indian government in 1947, and had signed a bilateral cultural agreement in 1952. In addition, the press frequently highlighted the achievements of athletes from these countries. For example, India's Lavy Pinto, the “fastest man in Asia,” who had just won the 100- and 200-m sprints at the first Asian Games in Delhi, appeared at the Berlin University Games. The press and IUS publications frequently mentioned India's field hockey team, unbeaten Olympic champions since 1928, whose popularity in Warsaw was such that they “set a record^124^” for the number of autographs signed before the final game. As the country was a “guest of honor^125^” at the Festival in Moscow, the field-hockey team was invited to take part in the demonstration of traditional sports, which was part of the unofficial program. Similarly to the traditional dance and music performance typical for WDFY and IUS events whatever their level, it probably aimed to valorize the cultural richness and authenticity of the faraway regions in front of the Western modern identity.

Only a few African nations competed in the 1951 University Games in Berlin, including Nigeria, which had formed a national Olympic committee in January 1951, 9 years before gaining independence^126^. It was preparing, alongside three other African countries, to take part in its first Olympic Games, the following year in Helsinki^127^. Numerous anecdotes in the press related the participation of Africans in the grassroots events. When the French authorities refused visa requests from two players in the football team of the Association of African Students in France^128^, “two black guys” from Martinique asked the judge for permission to make up the team^129^. Another African student, from Lati Tuakli technical college, whose nationality was not given, said every member of his country's Festival delegation intended to get the Festival Badge^130^.

**Figure 1:**
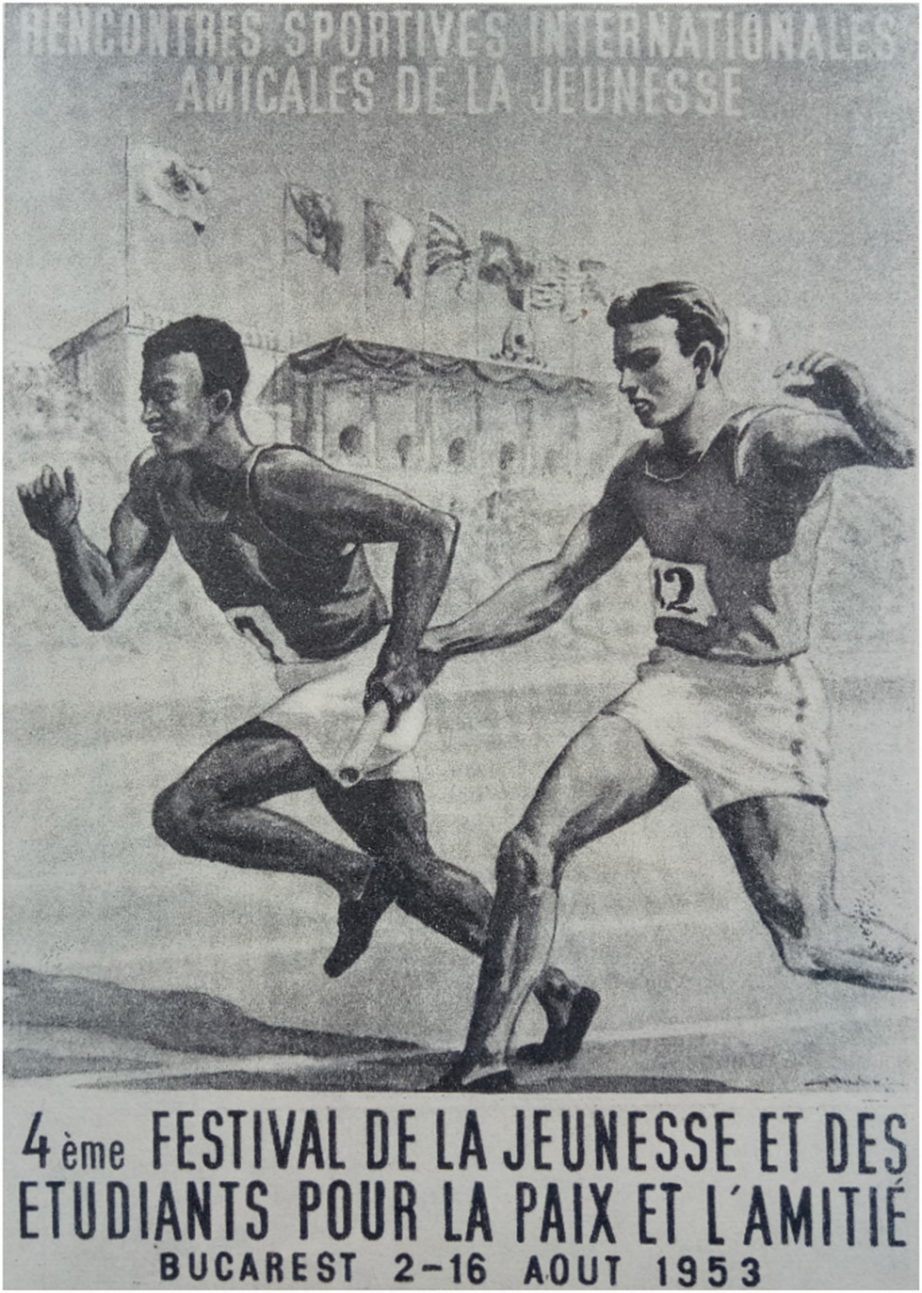
Billboard of the International Friendly Youth Sports Meetings, Bucharest, 1953. Source: Newspaper of the International Preparatory Committee, April 28–May 4, 1953, 3.

The attention paid to African nations increased greatly between 1953 and 1957, many of which were invited to the Festival in the context of growing relations with black power movements. This was a way of expressing solidarity with people struggling for independence and of rejecting racial discrimination, which was depicted in Festival posters. For example, the poster for the 1953 International Friendly Youth Games in Bucharest shows a white runner passing the baton to a black comrade (see Figure 1). Although the two runners obviously do not belong to the same team, as they are wearing different colored vests, the idea was to show that nationality, skin color, and performance are unimportant if they are both running for the same ideals. In the distance, the grandstands are topped by the flags of the “Big Four” winning countries from World War II plus communist China and Romania, the host country. Graphics for the Moscow festival also highlighted the participation of black athletes^131^. This focus on black athletes was part of the Soviet Union's desire to stress its antiracist, friendly and equal attitude, contrasting with a poor treatment of the black athletes by the USA.

Less coverage was given to participation by Middle Eastern countries in the sports events before 1955. Nevertheless, following the 1955 Friendly Games in Warsaw, the Soviet authorities published an interview in which the secretary of Egypt's Olympic committee, Ahmed Touny, highlighted the “exceptional goodwill shown via the friendly help given to weaker rivals^132^.” His comment was prompted by the assistance given to the Egyptian rowing team, which did not have its own coach, by a Polish rowing coach^133^, but it is probably no coincidence that it was reported at a time of escalating tension between Egypt and Israel, supported by France and the United Kingdom. Two years later, at the 1957 Moscow Festival, which took place just few months after the Suez Crisis, attention was drawn to an English table tennis player's sporting gesture of offering a glass of water, with a smile, to his Egyptian opponent^134^. Official publications and the Soviet press trumpeted such demonstrations of good sportsmanship as proof of young people's goodwill toward their foreign comrades regardless of the color of their skin or political leanings, and as contrasting with the imperialist behavior of the decadent empires. These examples alluded to the emerging unity of international socialist youth, where Global South could bring a great number of “potential friends” and supporters of the Soviet Union through their participation in the Festival^135^.

## Welcoming the World to the Soviet Capital: Moscow in the Role of International Sports Destination

Staging the III International Friendly Youth Games during the 1957 Moscow Festival gave the Soviet capital its first taste of being a world's new international sports destination. This third and final International Friendly Youth Games brought the world's youth to the capital of socialism in a spirit of peaceful coexistence, where the Festival had to promote a peaceful image of Kruschev's USSR^136^. It benefitted from the enduring euphoria generated by the country's performance at the Melbourne Olympics and took place at a time when the Soviet Union was beginning to open the borders to a larger number of visitors^137^. The warm welcome given to the first post-war influx of foreign tourists, who admired the gloss of a modern capital, was a good start in preparing the city for the Festival. After heading the medals table in Melbourne, at only its second Olympic Games, the Soviet Union was keen to flaunt its newfound sporting prestige to an international audience. In addition, Soviet sports officials had returned from Melbourne having learned a lot about organizing major sports events and sports infrastructure. Soviet authorities had decided not to bid for the 1964 Summer Olympics^138^; preferring to focus on this “mini Olympiad^139^” celebrating internationalism, peace, and friendship, which was organized under the auspices of the country's Olympic committee. In fact, there were strong parallels between the goals of these two events^140^. While the Olympic Games were becoming a battlefield between the United States and the Soviet Union, Moscow 1957 was presented as promoting peaceful coexistence and the progress of the Soviet sports system. Every means was used to demonstrate the Soviet regime's modernity and the progress it had made in physical education and sports training. Several dozen physical education and sports specialists (known as *fizorgs*) were trained to conduct fitness sessions for delegates and lead (accompanied by interpreters) guided tours of Moscow's sports venues. In addition, a “sports avenue” through the southwestern part of the city took curious visitors from Lenin Stadium to the city center. Stadiums, swimming pools, and sports halls in and around Moscow were used to stage a festival of international sport. The Moscow Friendly Games included 23 sports, 13 of which were open to women. This was a source of pride for the Soviet authorities because women were only allowed to compete in 7 of the 17 sports on the Olympic program. Thus, 671 women competed at the Friendly Games, compared with only 376 female athletes at the previous Olympics. The Moscow Friendly Games were truly a mass-participation event, involving nearly 4,000 “white, yellow, brown, and black^141^” athletes from 46 countries (similar numbers to previous editions of the Friendly Games)^142^. “Ambassadors of five continents^143^,” as *Sovetskij sport* called the athletes, paraded through the brand-new Lenin Stadium, which had opened in July 1956. “Covering 145 ha, with 1,000 permanent staff^144^,” the new stadium was the pride and joy of Moscow's authorities and would go on to host the 1973 Universiade and the 1980 Olympic Games. In 1957, it welcomed Olympic athletes from Australia, as well as other friends of peace from “faraway shores^145^.” In addition, several countries which had boycotted the Melbourne Olympics over the Suez Crisis took part in the Friendly Games, which featured 139 Olympic medalists from Melbourne, as-well as 17 world champions and world record holders^146^. Defeats of these champions were heralded in the Soviet as proof of the high level of competition.

Détente made Soviet-American sports friendship a favorite topic for the Soviet press. For example, the opening ceremony generated lines such as: “Friends meet again” and “Crowd applauded: Soviet delegation appeared. But when the American delegation came closer, Soviet [athletes] pounced on them to exchange cordial greetings^147^.” The athletes from English-speaking countries seemed also to be happy to come to Moscow, even though they were not necessarily supported in their home countries' governments or sports organizations. America's Parry O'Brian, who had won the shot-put competition in Melbourne accepted the invitation to the Friendly Games because they represented “a great contribution to strengthening Soviet-American sporting ties^148^,” adding that the Russians' hospitality exceeded anything he had ever seen. New Zealand's Norman Read, who had won Olympic gold in the 50-km walk, said: “the language of friendship does not need translators^149^.” This expression was all the more interesting because sport was also considered to be a universal language. In fact, scenes of famous athletes talking and laughing together showed that differences in language and culture did not prevent the Festival creating a relaxed and open atmosphere. Thus, at a time when the Soviet Union was becoming more open to the world, Moscow seized the opportunity to become the capital of international friendship and showed it was capable of hosting an event on the scale of the Olympic Games.

## Conclusion and Discussion

The World Festival of Youth and Students positioned itself as a champion of the values of peace, friendship and solidarity. The sports events held in conjunction with the Festival were a wonderful channel for spreading this message and created a new space of transnational cooperation. They provided the Soviet Union and the socialist states with an additional cultural diplomacy tool, whereby they helped to shape the Eastern bloc's relations with international sports organizations and other countries. By “overcoming Cold War the boundaries^150^,” the Festival purported to use sport to bring together young men and women in friendships that surpassed social, geographical, racial, and political differences. Students, office staff, and factory workers could now compete on the same track, for the same medals, regardless of their ability, and socialize outside the stadium. One objective of the Festival was to establish an additional space of repeated cross-border exchanges for the athletes and teams. The other consisted in moving away from the goal of sporting elitism and to encourage young people to take up sport, in line with the Soviet Union's desire to valorize and develop elite and grassroots sport (masterstvo and massovost, in Russian). The project looked to reform each individual at every level of society a to produce the “new socialist man.” Moreover, the idea of “sport for all” took on another dimension in the light of the rise of national independence movements in colonized countries. Young people from throughout the world could compete in the international competitions without having to wait for their country to set up an IOC-recognized Olympic committee. They also could obtain financial support for attending these celebrations of Soviet prowess, at which they were treated as guests.

The goal of Soviet sport ideologists was actually to offer a more inclusive model of multisport competitions, without explicitly competing with the Olympic institution or denigrating it. By moving away from the doctrine of sports elitism and holding the sports events in parallel with the Festival enabled the sports events to attract larger numbers of participants. Moreover, the Festival's biannual calendar, avoiding clashes with the quadrennial Olympics, meant that elite athletes, some of whom did not even have access to the Olympics, had a regular stage on which to compete. In order to legitimize and popularize the Games held in conjunction with the Festival, and to avoid accusations that they were encroaching on the Olympic Games, the organizers were invited and well-treated the representatives of the Western sports organizations. This was a key aspect of the Soviet Union's international sports crusade, whose aim was to gain the trust of Western organizations, including during the split in the university sports movement, from 1949 to 1957.

Of course, the absence of most of the West's top athletes helped ensure the Soviet Union and countries of the Eastern bloc won the vast majority of the medals, but their success in Melbourne showed that athletes from Eastern Europe could match, and often beat, their western counterparts. Unlike musical and artistic talent, sporting records provided quantifiable proof of the socialist system's achievements. Every victory by a Soviet athlete was projected as a triumph of communist values, so it was extremely important for Soviet cultural diplomacy to demonstrate success in a field where the country had long been isolated from the West. The Soviet Union undoubtedly hoped to continue organizing this forum of massive cross-cultural interaction but they were forced to abandon their plans following the political difficulties experienced by the next Festival, Vienna 1959. The other possible reason was the reunification of the international university sports movement under the auspices of FISU and the creation of the Universiade. The focus was now on gaining influence of the socialist countries within FISU. Consequently, future Festival's sports events risked to become much more modest affairs than those held during the “golden age” from 1949 to 1957.

The results cast a new light on a peculiar role of youth and sports as a channel of Soviet cultural diplomacy in Europe and the Global South during the Cold War. The paper also highlights the existence of two opposed models of international university sports promoted by the East and West, independently from the Olympic movement. In line with previous studies, the findings demonstrate the importance of the Festival sports competitions for t the internationalization of socialist sport and the democratization of international sport. Future research could thus focus on the national and regional perspectives as well as on the posterior development of sports at the Festival.

## Data Availability Statement

All datasets generated for this study are included in the article/supplementary material.

## Author Contributions

LL individually conducted the present research on all the stages.

## Conflict of Interest

The author declares that the research was conducted in the absence of any commercial or financial relationships that could be construed as a potential conflict of interest.

## Footnotes

^1^“12 dnei rekordov,” Molodež' mira (the Russian version of World Youth, the WFDY official magazine), 10, 1955, 38.

^2^Müller (2015).

^3^The International Preparatory Committee of each edition of the event was assisted by local sports institutions and its work was assisted and controlled by the Komsomol (All-Union Leninist Communist League of Youth), Communist Party of the Soviet Union (CPSU), and the corresponding Soviet ministries for each part of the program (for example, Committee for Physical Culture and Sports).

^4^Through the analysis of the previous scholarship Patryk Babiracki and Austin Jersild suggest that “socialist internationalism” represents the incarnation of the pre-war proletarian internationalism as a form of “egalitarian cooperation,” which is subject to the Soviet domination after the war. On the other hand, the term characterizes more informal exchanges and cross-cultural interaction fostered by organizations originated from the USSR. Festival seems to perfectly fit into this definition. Babyracki and Jersild (2016).

^5^Kott (2017).

^6^Defrance (2000).

^7^Koivunen (2013).

^8^Hoberman (1986). Cited by Koivunen, 55.

^9^Caute (2003). See Koivunen (2014).

^10^Keys (2006). Cited by Koivunen, P., Performing Peace and Friendship, 3–34.

^11^Koivunen, P., Performing Peace and Friendship, 52.

^12^Gillabert (2017). He also explains that these two terms are quite similar but arise from different historiographical traditions.

^13^Možaeva (2015).

^14^Clastres (2020). See also Murray (2012) and Rofe (2016).

^15^Kotek (1998).

^16^Schiller (2019).

^17^Parks (2013).

^18^Parks, J., 89.

^19^Riordan (1974). See also Riordan (1993).

^20^Parks (2014).

^21^Dufraisse (2019).

^22^Dufraisse (2012). See also O'Mahony (2006).

^23^Guerres mondiales et conflits contemporains 277(1).

^24^This difficulty related to the selective declassification, and the excessive use of “secret” label were also mentioned in Dullin (2003).

^25^Many publications have highlighted the link between sport and youth: “youth and sport: these two notions are inseparable,” Molodež' mira, 10, 1955, 38, and “it is impossible to imagine youth without sport,” Molodež' mira, 10, 1957, 18.

^26^Grant (2013).

^27^RGASPI (Russian State Archives of Socio-Political History), fund 17 (Central Committee of the Communist Party), inv.172, file 71, 167.

^28^Koivunen, P., Performing Peace and Friendship, 55.

^29^Activités de la Fédération mondiale de la Jeunesse démocratique, November 1949–August 1949, Paris, WFDY, 1949.

^30^“Meždunarodnyj festival' molodeži,” Sovetskij sport, 64(1356), August 12, 1947, 4.

^31^Sbetti (2020).

^32^IAAF and FIFA in 1946, FIBA in 1947, FIVB in 1948.

^33^Parks, J. op.cit.

^34^RGASPI, f.17, inv.172, f. file 42, 1-12.

^35^Named after the founder of the Sokol youth sports movement.

^36^“Tri festivalâ: Praga-Budapest-Berlin,” Fizkul'tura i sport, 8, 1951, 4.

^37^GARF (State Archives of Russian Federation), f.R7576, inv.1, f.592, 45–46, cited by Dufraisse, S. op.cit., 46.

^38^The first Men's volleyball world championship was organized in Czechoslovakia in 1949, while the International chess federation became one of the favorite partners of the USSR.

^39^See for example, Gounot (1998).

^40^“Sport. Olimpijskie igry i Triest,” Molodež mira, 1(7), 1948, 26–27. The article addressed Trieste's exclusion from the forthcoming Olympic Games in London due to the involvement of Italian fascists in the Olympic movement.

^41^Krieger and Duckworth (2020).

^42^Mayer to Edström, December 6, 1948, IOC Archives, IOC Presidents, Sigfrid Edström, A-P04/002.

^43^Edström to Mayer, December 9, 1948, IOC Archives, IOC Presidents, Sigfrid Edström, A-P04/002.

^44^Kotek, op.cit., 207–239.

^45^Koivunen, Performing Peace and Friendship, 67–74.

^46^Krieger and Duckworth (2020).

^47^Dufraisse, op.cit., 106.

^48^As the World University Games were managed by the IUS, they were object to all the political disagreements between within it. The tensions between Eastern and Western student unions reflected the changes of geopolitical situation, such as the launching of Marshall Plan, the outbreak of Korean War, and Tito-Stalin conflict.

^49^“S'iniziano oggi i Giochi Sportivi del V Festival della Gioventù,” *L'Unità* (the Italian communist party newspaper), 4 August 1953, 5.

^50^“Budapest vstrečaet gostej,” Sovetskij sport, 90(1590), 13 August 1949, 7.

^51^This decrease in participation was related to the West's increasing awareness of the Festival's political nature. See Kotek (2010).

^52^“Iz budapeštskogo dnevnika,” Fizkul'tura i sport, September 9, 1949, 20.

^53^”Résultats des X^è*mes*^ Jeux mondiaux universitaires d'été, Budapest, 14–21 août 1949.” Prague: IUS, 1949.

^54^Zubok (2014).

^55^Documents et décisions du 7^e^ Conseil annuel de la Fédération mondiale de la jeunesse démocratique. Berlin, 21–24 août 1951. Budapest: FMJD, 1951, 8.

^56^Perrin (1951).

^57^“Mir! Družba ! Mir!,” Fizkul'tura i sport, 8, 1951, 2–3.

^58^According to the Soviet sports classification.

^59^“Tri festivalâ: Praga-Budapest-Berlin,” op.cit.

^60^The numbers were provided in *Fizkul'tura i sport, Sovetskij sport, World Youth* and *L'Unità*.

^61^Navstreču festuvalû, *Sovetskij sport* 75(1878), June 28, 1951, 3.

^62^“Flag festivalâ,” Sovetskij sport, 90(1590), August 13, 1949, 3.

^63^Sbetti, N., op.cit.

^64^Mander, E. “Elevato contenuto tecnico degli XI Giuochi Universitairi,” L'Unità, August 9, 1951, 4.

^65^Riordan (1977).

^66^Parks, J., 73–106.

^67^International Organizing Committee of the XI World University Summer Games to Sigfrid Edström, 21 July 1951, IOC Archives, E-RE02-FISU/002.

^68^GARF, R7576, inv.2, f.662, 116.

^69^More specifically, Spain and German Democratic Republic.

^70^Carl Schneiter to Otto Mayer, May 12, 1955, IOC Archives, E-RE02-FISU/002.

^71^Carl Schneiter to Otto Mayer, August 1, 1955, IOC Archives, E-RE02-FISU/002.

^72^Riordan (1977).

^73^Otto Mayer to Carl Schneiter, May 16, 1955, IOC Archives, E-RE02-FISU/002.

^74^Several international federation leaders attended the Games in 1953: Gaston Mülleg (rowing), Roger Coulon (amateur wrestling), Paul Libaud (volleyball), and Bertil Sällfors (swimming). The Festival bulletin highlighted FINA's and the FIVB's satisfaction with the staging of the event and the event model. It also noted the enthusiasm of some European national federations (Austria, Italy, the Netherlands, etc.) for the event, Newspaper of the International Preparatory Committee, May 5–11, 1953, 1.

^75^Otto Mayer to Avery Brundage, August 5, 1953, IOC Archives, IOC Presidents, Avery Brundage, A-P05/016.

^76^Otto Mayer to Avery Brundage, June 30, 1953, IOC Archives, IOC Presidents, Avery Brundage, A-P05/016.

^77^Riordan (1977).

^78^Avery Brundage to Otto Mayer, July 8, 1953, IOC Archives, IOC Presidents, Avery Brundage, A-P05/016.

^79^Newspaper of the International Preparatory Committee, May 19–25, 1953, 1.

^80^Unknown author, Zatopek s'est imposé à Varsovie, Le Monde, August 4, 1955.

^81^Festival de Varsovie, 1955, Swiss Festival Committee leaflet, Archives cantonales vaudoises, PP798/55.

^82^“Sportivnye sostâzaniâ,” Komsomol'skaâ Pravda, August 1, 1953, 3.

^83^“Fereiro da Silva,” Molodež' mira, 10, 1957, 20.

^84^“Govorât sportsmeny,” Sovetskij sport, 95(1898), August 14, 1951, 7.

^85^Newspaper of the International Preparatory Committee, May 19–25, 1953, 1.

^86^“Listing as many countries as possible was part of the strategy to give the Festival legitimacy.” See Koivunen, P., Performing Peace and Friendship, 201.

^87^Koivunen (2016).

^88^Rider (2018).

^89^Parks, J., The Olympic Games, 89.

^90^“Navstrechu Olimpijskim igram v Melburne,” Molodež' mira, 11–12, 1955, 16.

^91^“Vo imâ olimpijskih idealov,” Molodež' mira, 10, 1955, 20–21.

^92^“12 dnei rekordov,” op.cit.

^93^“Eto sbližaet narody,” Fizkul'tura i sport, 9, 1955, 8.

^94^“Po 23 vidam,” Fizkul'tura i sport, 9, 1955, 7.

^95^Their participation was all the more important because the split in the student sports movement continued until 1959 and FISU's rival competitions took place at the same time of year.

^96^“Poslancy pâti materikov,” Sovetskij sport, 102(2838), 28 July 1957, 3.

^97^Dufraisse, Les héros du sport, 133–168.

^98^Sport at the Festival, Budapest: FMJD, 1957, 4–5.

^99^In the Soviet context this concept describes the new model of ideal citizen created by the state and incarnating specific values, beliefs and culture.

^100^“Vesti iz goroda festivalâ”, Komsomol'skaâ Pravda, 187(9285), August 9, 1955, 2.

^101^Newspaper of the International Preparatory Committee, April 20–27, 1953, 3.

^102^Sports at the Festival, op.cit.

^103^For example, Unione Italiana Sport Popolare (UISP) and Fédération sportive et gymnique du travail (FSGT), whose Cross de l'Humanité was a typical event. This is obviously related to the importance of the communist party in these countries.

^104^“Sport pour tous,” Newspaper of the International Preparatory Committee, March 15–31, 1955, 1.

^105^“Massovye sportivnye sorevnovaniâ,” Molodež' mira, 4, 1955, 24.

^106^GTO—“Gotov k trudu i oborone” (Ready for Labor and Defense). GTO was an unified system of physical training of all Soviet people with a special system of rewards according to the results.

^107^“Tri milliona učastnikov,” Molodež' mira, 10, 1955, 37.

^108^Rider (2018).

^109^Wishon (2016).

^110^“Festival' ûnosti i druž*by*,” Fizkul'tura i sport, 9, 1955, 4.

^111^“Beseda s prezidentom MSS J.Grohmanom,” Molodež' mira, 10, 1951, 17.

^112^“Vo imâ olimpijskih idealov,” op.cit.

^113^Berstein and Milza (2014).

^114^Fischer (2000).

^115^Paget (2004).

^116^“Na stadione Ujpest otkrylsâ Festival,” Sovetskij sport, 91(1591), August 14, 1949, 7.

^117^Parks, J., Welcoming the “Third World,” op.cit., 85.

^118^Charitas (2009).

^119^Brazil (105), Argentina (40), Cuba (25), Columbia (28), India (84), Indonesia (89), Nigeria (47), Iran (119), Liban (114).

^120^“Festival molodyh borcov za mir,” Sovetskij sport, 91(1591), August 14, 1949, 1.

^121^“Beseda s prezidentom MSS J.Grohmanom,” op.cit.

^122^“Festival' ûnosti i druž*by*,” op.cit.

^123^GARF, f.R7576, inv.2, f.666, 45.

^124^“Vo imâ olimpijskih idealov,” op.cit.

^125^Wishon (2016), op.cit.

^126^In January 1951, French IOC member François Pietri expressed his concerns about recognizing the Nigerian NOC. See Charitas, P., op. cit.

^127^The other three were Gold Coast (Ghana), the Union of South Africa, and Egypt.

^128^“S dalekih beregov,” Sovetskij sport, 104(2840), July 30, 1957, 7. This episode is also mentioned in Dieng (2011).

^129^“Na stadionah družby,” Molodež' mira, 10, 1957, 18.

^130^“Massovye sportivnye sorevnovaniâ,” op.cit.

^131^The cover of Fizkul'tura i sport, 7, 1957.

^132^“Sportsmen from Arab countries to Warsaw,” Newspaper of the International Preparatory Committee, 16–31 May 1955, 2.

^133^“Festival' ûnosti i družby,” op.cit.

^134^“Cem nehorošy igry,” Molodež' mira, 11, 1957, 8–9.

^135^Koivunen (2016).

^136^Koivunen (2009). Some other events organized in the USSR during Détente pursued similar goals: for example, the Congress of Sports Medicine (1958) or especially the World Youth Forum (1961).

^137^Salmon (2006).

^138^Dufraisse (2020).

^139^“Simfoniâ družby,” Fizkul'tura i sport, August 8, 1957, 15.

^140^See García and Magnúsdóttir (2019) and Redihan (2017).

^141^“Un coup d'oeil sur les compétitions sportives,” Étudiants du monde (French version of World Student News, the IUS official magazine), 8–9, 1956, 15.

^142^Four thousand and three hundred participants from 54 countries in Bucharest and between 4,000 and 5,000 athletes from 42 countries in Warsaw.

^143^“Poslancy pâti materikov,” op.cit.

^144^“Un coup d'œil,” op.cit.

^145^“S dalekih beregov,” op.cit.

^146^“Vokrug družeskih igr,” Fizkul'tura i sport, 9, 1957, 13.

^147^“Simfoniâ družby,” Fizkul'tura i sport, 6, 1957,

^148^“Gotovimsâ, priedem,” Fizkul'tura i sport, 6, 1957, 4.

^149^“V razgovore s druz'âmi,” Fizkul'tura i sport, 9, 1957, 14.

^150^Koivunen (2010).
